# Directed Binding of Gliding Bacterium, *Mycoplasma mobile*, Shown by Detachment Force and Bond Lifetime

**DOI:** 10.1128/mBio.00455-16

**Published:** 2016-06-28

**Authors:** Akihiro Tanaka, Daisuke Nakane, Masaki Mizutani, Takayuki Nishizaka, Makoto Miyata

**Affiliations:** a Graduate School of Science, Osaka City University, Sumiyoshi-ku, Osaka, Japan; b Department of Physics, Faculty of Science, Gakushuin University, Toshima-ku, Tokyo, Japan; c The OCU Advanced Research Institute for Natural Science and Technology (OCARINA), Osaka City University, Sumiyoshi-ku, Osaka, Japan

## Abstract

*Mycoplasma mobile*, a fish-pathogenic bacterium, features a protrusion that enables it to glide smoothly on solid surfaces at a velocity of up to 4.5 µm s^−1^ in the direction of the protrusion. *M. mobile* glides by a repeated catch-pull-release of sialylated oligosaccharides fixed on a solid surface by hundreds of 50-nm flexible “legs” sticking out from the protrusion. This gliding mechanism may be explained by a possible directed binding of each leg with sialylated oligosaccharides, by which the leg can be detached more easily forward than backward. In the present study, we used a polystyrene bead held by optical tweezers to detach a starved cell at rest from a glass surface coated with sialylated oligosaccharides and concluded that the detachment force forward is 1.6- to 1.8-fold less than that backward, which may be linked to a catch bond-like behavior of the cell. These results suggest that this directed binding has a critical role in the gliding mechanism.

## IMPORTANCE

*Mycoplasma* species are the smallest bacteria and are parasitic and occasionally commensal, as represented by *Mycoplasma pneumoniae*, which causes so-called “walking pneumonia” in humans. Dozens of species glide on host tissues, always in the direction of the characteristic cellular protrusion, by novel mechanisms. The fastest species, *Mycoplasma mobile*, catches, pulls, and releases sialylated oligosaccharides (SOs), which are common targets among influenza viruses, by means of a specific receptor based on the energy of ATP hydrolysis. Here, force measurements made with optical tweezers revealed that the force required to detach a cell from SOs is smaller forward than backward along the gliding direction. The directed binding should be a clue to elucidate this novel motility mechanism.

## INTRODUCTION

**FIG 1:**
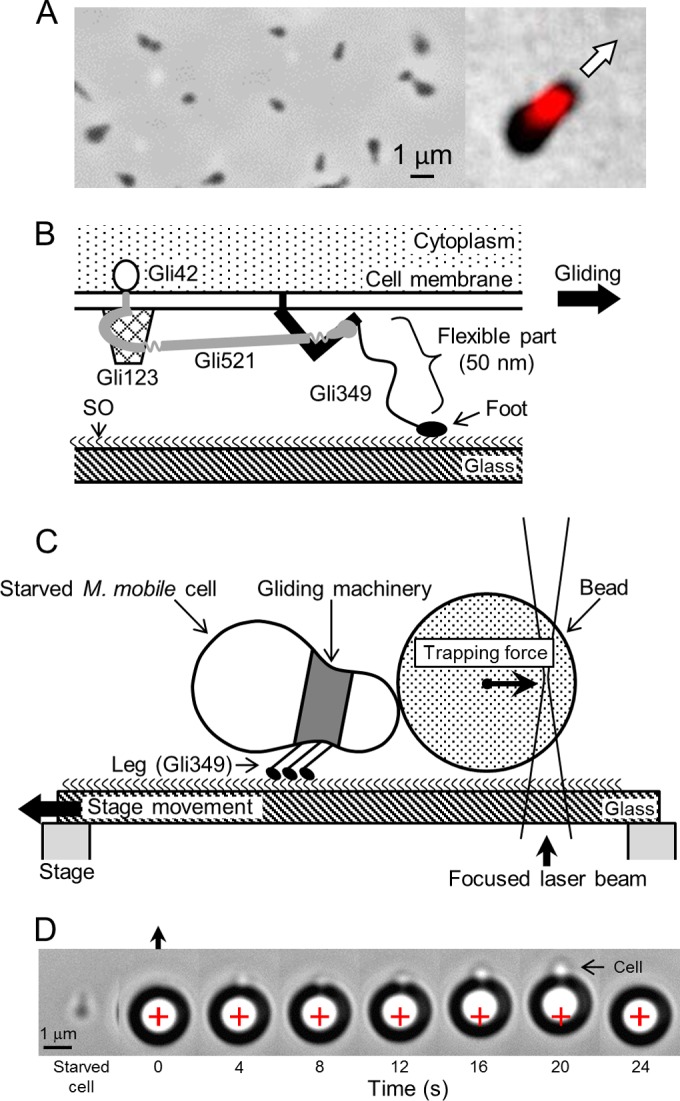
Design of experiment to measure force of detachment of *Mycoplasma mobile* cells from SOs. (A) Images of *M. mobile* cells. Left, single phase-contrast image from movie; right, merged image from phase-contrast and immunofluorescence microscopy. The gliding machinery is labeled in red. The cell glides in the direction of the arrow. (B) Schematic illustration of unit of the gliding machinery. The unit is likely composed of four proteins, Gli42, Gli123, Gli349, and Gli521. The leg protein, Gli349, anchored to the cell membrane at the N terminus, is composed of two rigid rods, a flexible part, and a foot, and catches and pulls SOs fixed on the solid surface through the foot. The movements generated by ATP hydrolysis are likely transmitted through Gli521 to the Gli349 molecules. This schematic is based on the molecular shapes, genetic and immunological data, and other information determined to date (3, 6, 16, 36). (C) Experimental design. *M. mobile* cells, surface labeled with biotin, were starved so as not to glide. A polystyrene bead, 1.5 µm in diameter and coated with avidin, was bound to a cell using optical tweezers. The bead was bound at either the front or the back end of the cell. The glass was moved at a constant rate by an actuator attached to the microscope stage, forward or backward relative to the cell attached to the trapped bead. These illustrations are not to scale. (D) Consecutive movie images of the detachment of an *M. mobile* cell from the glass surface by displacement at a constant speed. A starved, immobile *M. mobile* cell that had been gliding in the direction shown by the black arrow at the top was attached to a bead (large black ring with white center) manipulated by optical tweezers. The sample stage on which the cell was bound was moved in the direction of the black arrow at a speed of 160 nm s^−1^. The center of the bead was biased from the center of the trap (marked by a red cross) by the force exerted through the cell. The cell (marked by an arrow) was partly visible as a white spot when it was stretched by the pulling force. After 20 s, the cell had become detached from the glass, and the attached bead returned to the trap center at 24 s.

*Mycoplasma* species, the smallest bacteria, are parasitic and occasionally commensal, with small genomes that lack genes encoding a peptidoglycan layer (1, 2). Dozens of *Mycoplasma* species form protrusions (3–7), such as the so-called head and neck in *Mycoplasma mobile* and the attachment organelle in *Mycoplasma pneumoniae* (4, 8), a human pathogen that was epidemic a few years ago (9). On solid surfaces, these species exhibit gliding motility in the direction of the protrusion; this motility appears to be involved in the parasitism of mycoplasmas (7, 10). Interestingly, mycoplasmas have no flagella or pili, and their genomes contain no genes related to known mechanisms of bacterial motility. In addition, no homologs of motor proteins that are common in eukaryotic motility have been found (11, 12). *M. mobile*, isolated from the gills of a freshwater fish, is a fast-gliding *Mycoplasma* (Fig. 1A; see also Movie S1 in the supplemental material) (13–17). It glides smoothly and continuously on glass at an average speed of 2.0 to 4.5 µm s^−1^, or three to seven times the length of the cell per second, exerting a maximum force of about 30 pN (15). In quick-freeze replica electron microscopy, 50-nm-long leglike structures are observed to stick out from the base of the protrusion and attach to the solid surface at their distal ends (18). We have identified huge proteins, Gli123, Gli349, and Gli521, with masses of 123, 349, and 521 kDa, respectively, that localize on the machinery surface (Fig. 1B) and are involved in this gliding mechanism (19–22), and we have visualized the molecular shapes of these isolated proteins (23–26). We have identified the internal structure of the machinery, named the “jellyfish” structure, which consists of a bell shape at the cell front connected by dozens of tentacular strands comprised of 20-nm particles at 30-nm intervals, as well as the component proteins of the machinery (27, 28), the direct energy source used (29, 30), the direct binding target (31–33), and the unitary steps of the movements (34). On the basis of these results, we proposed a working model, called the centipede or power-stroke model, where cells are propelled by flexible “legs” composed of Gli349 that, through repeated cycles, catch, pull, and release sialylated oligosaccharides (SOs) fixed on the glass surface via the distal “feet” (3, 5–7, 35). This working model is based on an assumption that the leg, plausibly Gli349, detaches more easily when it is pulled forward than backward (3, 6, 35). However, this assumption has not been examined experimentally. In the present study, we measured the force and the cell interaction lifetime for detachment of starved *M. mobile* cells from SOs fixed on a glass surface and concluded that detachment can occur more easily forward than backward.

## RESULTS

### Experimental design.

To examine the directionality of binding, we designed an experiment, illustrated in Fig. 1C, in which an *M. mobile* cell stopped by starvation on a glass surface covered with SOs was attached to a 1.5-µm-diameter bead at the front or back end of the cell through avidin-biotin interaction. Then, the cell was detached from the surface by using optical tweezers to pull the bead. The detachment process was analyzed in two ways. In the first analysis, the cell was pulled with a constant speed and the force at detachment was analyzed. In the second analysis, the cells were pulled rapidly for various distances and the time required for detachment was analyzed as the cell interaction lifetime.

### Measuring detachment forces.

*M. mobile* cells, biotinylated and suspended in phosphate-buffered saline (PBS), were inserted into a tunnel chamber coated with horse serum, which contained proteins conjugated with SOs. Most cells inserted into the tunnel attached to the glass surface, glided at a speed of 0.31 ± 0.30 µm s^−1^, and stopped completely after 100 to 600 min at room temperature (RT). This stop was likely caused simply by starvation, because the replacement of the PBS in the tunnel with PBS containing 20 mM glucose caused the cells to restart gliding in 2 min. The cells likely bound to the glass mostly through the bonds between Gli349 and SOs under the experimental conditions, because no cells bound to the glass in the presence of 1 mM free sialyllactose, an SO structure, and no cells of a nonbinding mutant with a single amino acid substitution in Gli349 bound to the glass, as previously reported (16, 31–33, 36). Next, we added to the tunnel 1.5-µm-diameter beads coated with avidin. A bead was trapped by optical tweezers and attached to the front or the back end of a cell by moving the stage of the microscope so that the cell and the bead made contact (see Movie S2 in the supplemental material). The stage was then moved in the direction of the front or back of the cell at a speed of 160 nm s^−1^ while the bead was kept near the trap center. The bead moved from the trap center as the stage made a small horizontal movement and was carried back to the trap center after the cell detached from the glass (Fig. 1D). The beads likely bound to the cells through the avidin-biotin interaction, because the beads pulled the cells with a frequency of less than 20% and a force of less than 40 pN when they were attached to the cells without the avidin coating. When we examined wild-type cells, we found that detachment was sometimes not achieved due to the large detachment forces. Therefore, we used a mutant strain whose binding and gliding are half those of the wild-type strain (see Movie S3); the strain was constructed by replacing serine with arginine at the 859th amino acid in the whole 4,727-amino-acid length of the Gli521 protein [*gli521*(S859R)], which is essential for gliding (36).

### Tracing a detachment event.

**FIG 2:**
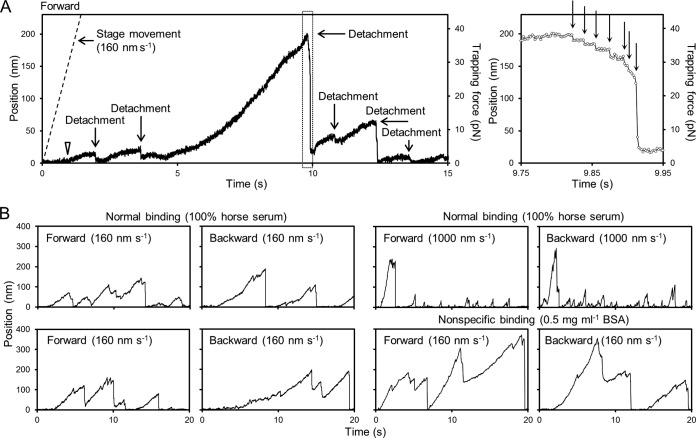
Distances between bead and trap centers when the *M. mobile* cell was pulled by the movement of the stage at a constant speed. (A) Left, the distance between the bead and trap centers was traced through time. The cell, bound to the bead at the front, was pulled forward by moving the stage. The time point when the bead started to move from the trap center is marked by an open triangle. The time points of detachment are marked. The force exerted on the bead was calculated from the distance between the bead and trap centers. The speed of stage movement, 160 nm s^−1^, is shown as a dashed line. Right, magnified image of dotted box in the left panel. The detachment events are marked by arrows. (B) Bead traces under various conditions. The four left and two upper right graphs show the traces of the gliding *gli521*(S859R) mutant, whose binding and gliding are reduced by half from those of the wild-type strain. The two bottom right graphs show the gliding traces of the same strain adsorbed to the glass without SOs. The force direction and moving speeds of the stage are indicated in each graph.

We traced the movement of the bead center with the moving stage for 30 s, as partly shown in Fig. 2, and calculated the force exerted on the bead from the distance between the bead and the trap center. In this trace, the bead did not move from the trap center until the stage had been moving for 0.8 s at a speed of 160 nm s^−1^. After 0.8 s, the bead center was biased from the trap center. The bead moved at speed of 14.5 to 55.4 nm s^−1^ because the cell body was elongated and tension was applied.

The small and large displacements of the bead toward the trap center observed at 2.0, 3.6, 9.8, 10.8, 12.3, and 13.6 s were marked as detachment events that were likely caused by the detachment of individual legs from SOs. Four additional traces are shown in Fig. 2B. Next, to analyze the detachment events, we traced the bead movements in detail and found that the process could be resolved into multiple events, as shown on the right in Fig. 2A. We judged a bead movement to be a detachment when it overcame 1.5 times the repetitive noises ranging from 0 to 5.0 nm and the binding lasted longer than 0.4 s just before the detachment. The forces for detachments occurring less than 0.4 s after the previous detachment were not compiled, because in such cases, the force may have been transient, unlike the case with the force applied to the first detachment.

### Detachment forces under various conditions.

**FIG 3:**
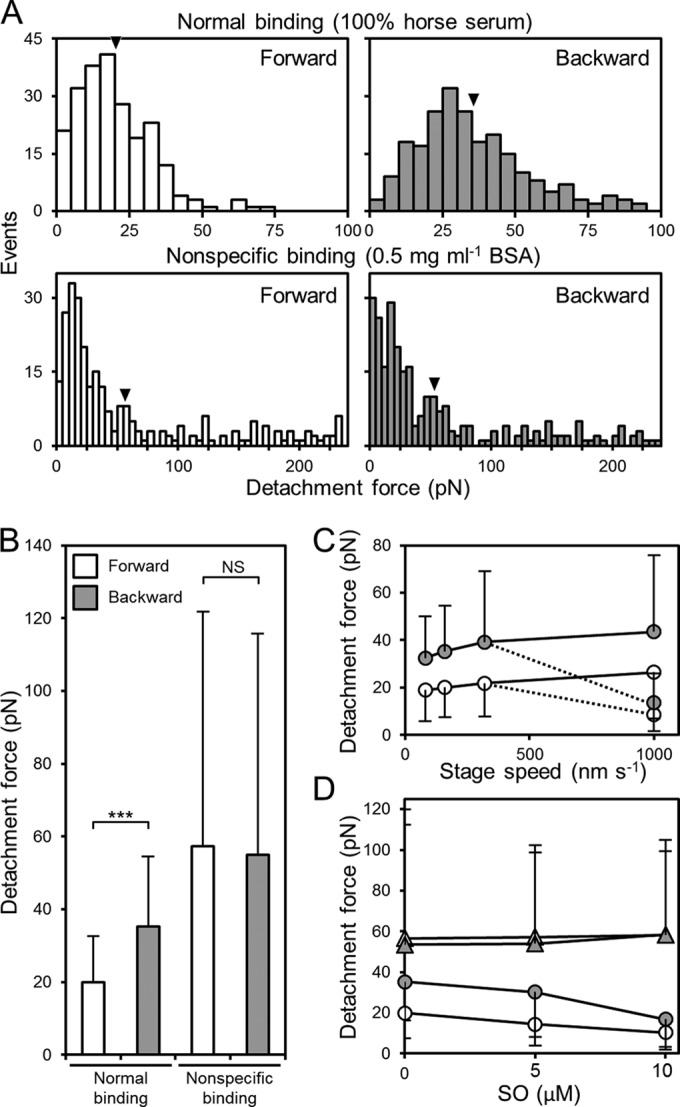
Distribution of forward and backward detachment forces. (A) Distributions of detachment forces. The numbers of forward and backward detachment events at different forces resulting from pulling at a speed of 160 nm s^−1^ are presented. Different coatings were used on the glass, as indicated. The mean in each panel is shown by a filled triangle. (B) Detachment forces of normal and nonspecific binding. ***, *P* < 0.001; NS, nonsignificant at a *P* value of >0.05 (Mann-Whitney *U* test). (C) Detachment forces under various speeds of the stage. Forward and backward detachment forces are shown by open and shaded circles, respectively. At 1,000 nm s^−1^, the initial and subsequent detachments are marked by solid and dotted lines, respectively. (D) Detachment force in the presence of free SO, GSC-28. Normal and nonspecific binding are presented by circles and triangles, respectively, and forward and backward detachments are presented by open and shaded symbols, respectively. The stage was moved at 160 nm s^−1^. Error bars for SD are shown only on the upper or lower side in panels B, C, and D.

The bead positions relative to the trap center at detachment were distributed with mean distances and standard deviations of 103 ± 63 (*n* = 227, 6 cell preparations) and 176 ± 84 nm (*n* = 229, 6 cell preparations) for forward and backward movement, respectively. The mean displacements where the bead started to move from the trap center, 36 ± 33 nm (*n* = 12), did not differ significantly between forward and backward movement. The forces required for detachment by pulling the cell at a rate of 160 nm s^−1^ were calculated to be 20.0 ± 12.6 and 35.3 ± 19.1 pN for forward and backward movement, respectively, showing that cells binding to SOs on glass are detached more easily forward than backward (Fig. 3A and B). Next, we measured the detachment force with various speeds of the stage, as follows: 80, 160, 320, and 1,000 nm s^−1^ (Fig. 2B and 3C; see also Fig. S1 in the supplemental material). In the range of 80 to 320 nm s^−1^, the detachment forces increased slightly with the stage speeds, in keeping with the idea that the forward force is less than the backward, with ratios in the 1.7- to 1.8-fold range (*n* = 942). At 1,000 nm s^−1^, the force changed significantly from the initial to the subsequent detachments. Two typical traces are shown in Fig. 2B, upper right. The directionality in binding was detected as well. The average forces for forward detachment were 26.4 ± 19.7 (*n* = 24) and 8.7 ± 7.1 pN (*n =* 119) for the initial and subsequent detachment events, respectively, and those for backward detachment were 43.6 ± 32.3 (*n* = 18) and 13.5 ± 12.3 pN (*n =* 67) for the initial and subsequent events, respectively (Fig. 3C). The increase in detachment force with the stage speed was unlikely to have been caused by the liquid flow applied to the bead in the stage movement, because the applied force was estimated to be less than 0.015 pN from Stokes’ law, even at the fastest stage speed, 1,000 nm s^−1^. The faster stage movement may cause greater displacement of the beads before the cell detachment, resulting in the apparently larger detachment forces (37).

It is possible that the polarized cell shape is what caused the difference between the forward and backward detachment forces. Therefore, as a control, we measured the detachment forces of *M. mobile* cells bound to glass with an interaction other than that between Gli349 and SOs. Generally, washed *M. mobile* cells adsorb tightly to washed glass surfaces (30, 32, 33). In the present study, we used this method to bond the cells to the glass. To adjust the detachment force so that it was similar to the force in normal binding, we treated the washed glass with 0.5 mg ml^−1^ bovine serum albumin (BSA) for 1 min at RT. The typical traces are shown in Fig. 2B, bottom right. In the control experiments, the forward and backward detachment forces were calculated as 56.5 ± 63.3 and 53.6 ± 58.7 pN, respectively, at a pulling speed of 160 nm s^−1^ (Fig. 3A and B). To confirm that the detachment occurs between the feet and the SOs, the detachment force was measured in the presence of a free SO, GSC-28, the most effective compound for *M. mobile* detachment in SOs examined so far (Fig. 3D) (32). The detachment force decreased as the concentration of GSC-28 ranged from 0 to 0.01 mM, suggesting that the legs caught the free GSC-28 instead of the SOs fixed on the solid surface. In the presence of 0.02 mM GSC-28, the number of detached cells in the buffer increased with time and interfered with the bead trapping in 60 s. These results show that the detachments measured here occurred between the feet and the fixed SOs.

### Cell interaction lifetime.

**FIG 4:**
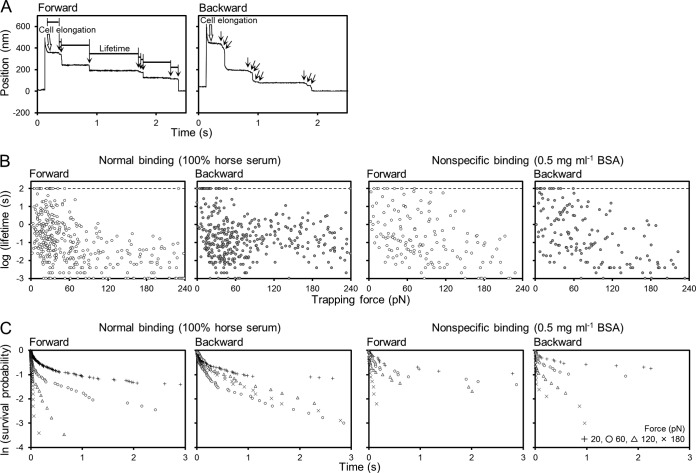
Bond lifetimes of cells pulled forward or backward 50 to 1,000 nm at 2 mm s^−1^. (A) Representative traces from 0.12 s of movement. The bead position is plotted to time. The time points when the bond was ruptured are marked by arrows. The rest time of the bead is marked “Lifetime.” The displacement caused by cell elongation is marked by the open arrow labeled “Cell elongation.” (B) Forward and backward bond lifetimes of normal and nonspecific binding. The lifetimes are plotted as a function of force. The circles plotted on the dashed line represent cells that did not detach from the glass within 100 s. (C) Bond survival probability as a function of time at various forces. Data from the ranges of 5 to 35, 45 to 75, 105 to 135, and 165 to 195 pN are summarized and plotted as 20, 60, 120, and 180 pN, respectively, as shown in the key at the bottom right.

Next, we compared the directionality of binding by cell interaction lifetime. A bead 1.5 µm in diameter was attached to the front or the back end of a starved *gli521*(S859R) mutant cell. The stage was moved at 2.0 mm s^−1^ to various displacements ranging from 50 to 1,000 nm forward or backward from a cell, with the bead trapped by optical tweezers. A typical trace of bead displacement is shown in Fig. 4A. The bead moved rapidly from the trap center, moved back slightly, and then stopped. The slight movement just after the stage displacement was likely caused by cell elongation, as Movie S4 in the supplemental material suggests. During the rest time, a pulling force was exerted on the bead attached to the cell. After various time periods, the bead moved to the trap center as a result of the detachment of the cell from the SOs on the glass surface. We traced the bead movements for 120 s and measured the duration from stop to detachment as the “cell interaction lifetime.” The step-by-step detachment was also observed here, but each lifetime was measured as an individual event, because the force should be exerted on the bond in a manner similar to that for the first detachment in the measurement of lifetime. As a control, we measured the interaction lifetimes of cells nonspecifically bound to glass by the adsorption procedure described above and used in the experiments whose results are shown in Fig. 2B, bottom right, and Fig. 3A, bottom. The cell interaction lifetimes decreased as the force increased to 240 pN, although some fraction of the lifetimes could not be determined by the smaller trapping forces (Fig. 4B), and there was no significant difference between the lifetimes of forward detachments (36 runs, 149 events, 3 cell preparations) and backward detachments (35 runs, 150 events, 3 cell preparations), suggesting that the polarized cell shape did not affect the interaction lifetime in backward detachment. The lifetimes of normal binding decreased accordingly as the forward pulling force increased in the range of 0 to 240 pN (89 runs, 387 events, 5 cell preparations). On the other hand, when the cell was pulled backward, the interaction lifetime did not decrease in accordance with the tension (92 runs, 372 events, 5 cell preparations). The cell interaction lifetime tended to decrease as the force increased at forces lower than 60 pN and then increase as the force increased to 120 pN, after which it decreased again (Fig. 4B). Therefore, we analyzed the cell interaction lifetime as bond survival probability, because this analysis is not affected by the failure in measurements of lifetime at smaller tensions. The bond survival probabilities between the cells and SOs at various forces decreased with time, as shown by the results in Fig. 4C. The rate of decrease depended on the force exerted on the bead in the forward detachment with normal binding and in both directions of detachment with nonspecific binding, but in the backward detachment with normal binding, an inversion was observed in the range of 60 to 120 pN.

## DISCUSSION

### Directed binding observed in *M. mobile*.

**FIG 5:**
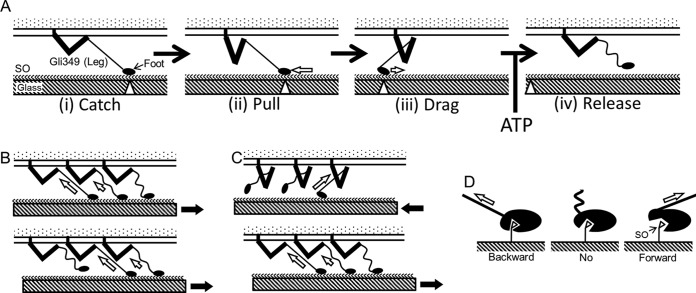
Schematics for gliding mechanism (A) and detachment of feet from SOs by pulling force (B, C, and D). (A) Centipede model to explain gliding mechanism by repetitive cycle through stages i to iv. The open triangles and open arrows indicate the attachment position on the glass and the direction of force exerted on the foot, respectively. The directed binding shown in the present study supports this model. (i) Catch: the foot at the end of leg (Gli349) catches an SO on the solid surface by chance. (ii) Pull: the tension applied to the leg from the front may trigger a conformational change in Gli349 by movements transmitted through other surface proteins, causing the pull. (iii) Drag: cell movement occurring as a result of the action of other legs pulls the “after stroke” foot, generating drag force. (iv) Release: the foot is removed in the forward direction and becomes ready to catch the next SO. The conversion from stage iii to iv may be coupled with ATP binding to the machinery. (B) Ordered detachment of three feet in a cell detachment. Each foot catches an SO molecule at a different position relative to the SO layer (top). The glass is moved in the direction of the black arrow. The foot that is pulled at the greatest tension, presented by the larger open arrow, becomes detached, resulting in a small displacement of the cell (bottom). The greatest tension is then applied to the next leg. (C) Conceptual model for directed binding caused by the number of legs contributing to the detachment force. The glass is moved in the direction of the black arrow, and the legs support the tension as shown by open arrows. The detachment force is caused by a single leg in the case of forward detachment (top) but by both a main leg and a supporting leg in the case of backward detachment (bottom). (D) Conceptual model for directed binding by the “ratcheting” characteristic of the foot. The states of the foot with forward (right), no (center), and backward (left) tension are illustrated. The bond is simply weakened by the forward tension but stimulated by the backward tension. In gliding, the state progresses from catch to release from left to right.

In the present study, we detected directionality in the binding of *M. mobile* cells to SOs through two different types of measurements, showing that the cells are removed forward more easily than backward. Previously, we showed that *M. mobile* gliding can be explained by a model, called the centipede model, where the feet of the *M. mobile* cell repeatedly catch, pull, drag, and release SOs, based on the energy from ATP hydrolysis (Fig. 5A) (3, 6, 35). This model is based on an assumption that the foot can be removed more easily in the forward direction than in the backward direction, which has been supported in the present study. Regarding the coupling of ATPase and repeated binding, new ATP molecules may be required for the removal of a foot from an SO, because *Mycoplasma* cells remained on SOs when ATP was suddenly depleted in the gliding ghost experiments (29, 34). Possibly, the bound ATP may be converted to ADP and P_i_ before the foot catches, and the ADP may be released before the foot releases. However, further studies are necessary to determine the details. Interestingly, two proteins, MMOB1660 and MMOB1670, in the internal jellyfish structure of the gliding machinery show high similarity to the α- and β-subunits, respectively, of F_1_-ATPase, the catalytic subunit of proton pumps, suggesting that this gliding mechanism may be developed from F_1_-ATPase (3, 27, 28, 38).

The manner of binding against the backward force may be considered a catch bond-like cell behavior, where receptor-ligand bonds are strengthened by tensile mechanical force (39, 40), because the bond survival probability decreased according to the forward tension but did not decrease in a similar way according to the backward tension and gave a lower rate, around 180 pN rather than 60 pN (Fig. 4).

### Mechanism for directed binding.

How, then, is directionality produced? Our previous studies suggested that hundreds of flexible legs sticking out around the gliding machinery have the ability to catch SOs (18, 21, 31, 32, 36). A leg is composed of a large protein, Gli349, consisting of 3,183 amino acids. The isolated Gli349 molecule has the binding activity to SOs and is shaped like a flexible musical eighth note, 100 nm long, including a transmembrane segment at its N terminus that is connected by two rather thick and rigid rods of about 40 nm in total (Fig. 1B) (24, 25, 41). The foot, the probable receptor domain for SOs (36), is connected to the rigid rods through a flexible thin string, about 50 nm long, which is composed of weak repeats of about 100 amino acids (26). Gli349 molecules are likely aligned on the machinery’s surface because of their high density in the limited space (20) and are likely directed by the internal structure along the cell axis (27, 28). The other surface proteins, Gli521 and Gli123, may modify the binding activity of Gli349, because some mutations and monoclonal antibodies to the other surface proteins modify the binding activity of cells (36).

The results of the present study suggest that the cell catches SOs through multiple legs, because cells were detached step by step in the pulling process, as shown in the right graph of Fig. 2A, and 4A. In such situations, the cell may have bound to the solid surface through legs under different tensions (Fig. 5B). When a bond with the highest tension was ruptured, a small displacement occurred, and the displacement stopped when another bond attained a similar tension. The formation of a second bond may have taken some time, because the tensions generated in the subsequent binding were obviously smaller than those generated in the initial one, when the cells were pulled at a high speed, 1,000 nm s^−1^ (Fig. 2B and 3C; see also Fig. S1 in the supplemental material). Although the rupture may have occurred in the bond with the highest tension, other bonds may have partly supported the tension at the initial displacement. Considering that hundreds of legs are aligned all around the gliding machinery (20, 21), the directed binding may be explained by assuming that more legs can participate in the binding in the backward tension than in the forward one. The putative difference in the distances of leg extension between forward and backward directions may cause such a difference (Fig. 5C).

However, the present results cannot negate the possibility that the directionality may be caused by a possible ratcheting characteristic of the foot part itself. *M. mobile* cells likely recognize the structures rather than only the negative charge, a common feature of SOs, as in a lock-and-key manner, because small modifications in the structures of SOs drastically affected the binding affinity for SOs even when the negative charge of sialic acid was maintained (31–33). The specific manner of recognizing SOs may cause directed binding at the foot (Fig. 5D). In this scenario, as the foot is connected to the C-terminal end of the flexible part of the Gli349 molecule, the foot should be directed along the cell axis through additional supports from other structures. Possibly other proteins essential for gliding, including Gli521 and Gli123, may play this role.

### Directionality and catch bond in motility.

In a catch bond generally, the distortion of a receptor caused by tension strengthens the interaction between receptor and ligand (39, 40). Today, many types of cells are known to use catch bonds to adhere to tissue surfaces, including uropathogenic *E. coli*, leukocytes, and platelets (42). Leucocytes roll on vessel surfaces by using catch bonds between their receptors and SOs (43). *M. mobile* may add energy from ATP to a so-called “rolling mechanism” and achieve active gliding (3, 5–7, 35, 41). *M. pneumoniae*, a cause of human pneumonia, is distantly related to *M. mobile* (44). This species also glides on solid surfaces, including host tissues, but the proteins essential for gliding do not share amino acid sequences with those *M. mobile* uses for gliding (3-7). However, both species form the machinery as a protrusion at a cell pole and glide in that direction via repeated binding of SOs on host tissue surfaces by the leg protein. The directed binding caused by a catch bond may be involved in *M. pneumoniae* gliding also.

The repetitive catch, pull, and release driven by ATP energy is common with more conventional motor proteins, such as myosin, dynein, and kinesin. The directed binding of motor proteins on rails such as actin and tubulin filaments has been discussed for many years (45–47). In the cases of the motor proteins, the rails have directionality, which is apparently different from the case of *Mycoplasma*, which glides on the layer of randomly aligned SOs. Although catch bonds have been studied mainly for sugar receptors, recent studies suggested catch bond properties for the motor proteins (45, 46). Even though the gliding machinery of *Mycoplasma* originated from other systems than the conventional motor proteins, it may achieve the common mechanism, i.e., repetitive binding driven by ATP energy, by convergent evolution. It is notable that the ratio of detachment force for *M. mobile* cells, 1.8, is comparable to those of 1.45 and 2.0 to 2.3 for kinesin and cytoplasmic dynein, respectively (47, 48), although our system should contain more unknown factors.

## MATERIALS AND METHODS

### Strains and culture conditions.

The wild-type and mutant strains of *M. mobile* 163K (ATCC 43663) were grown in Aluotto medium at 25°C (16, 36, 49). Cells were cultured to reach an optical density at 600 nm of 0.06. The mutant strain carrying a *gli521*(S859R) mutation was reported previously (36).

### Surface modifications of *M. mobile* cells and polystyrene beads.

The cells were biotinylated by using succinimidyl-6′-(biotinamido)-6-hexanamido hexanoate (Ez-Link Sulfo-NHS-LC-LC-biotin; Thermo Scientific, Waltham, MA) (50). Cultured cells were washed twice with PBS consisting of 60 mM sodium phosphate (pH 7.3) and 54.4 mM NaCl, suspended in 1.0 mM sulfo-NHS-LC-LC-biotin in PBS, and kept for 30 min at RT. To remove the excess biotin reagent, the cells were washed twice with PBS. Polystyrene beads (1.5 µm in diameter; Polysciences, Warrington, PA) were coated with avidin (Sigma-Aldrich, St. Louis, MO) according to the protocol of the protein coupling kit for COOH microparticles (Polysciences) and suspended to 5 × 10^4^ particles ml^−1^.

### Force measurements by optical tweezers.

The *M. mobile gli521*(S859R) strain suspension was inserted into a tunnel chamber (3-mm interior width, 22-mm length, 40-µm wall thickness). The tunnel chamber was constructed using two coverslips assembled with double-sided tape and was precoated with 100% horse serum or 0.5 mg ml^−1^ BSA for 1 min (30, 32, 33, 38). Both ends of the tunnel were sealed with nail polish if necessary.

The optical tweezers were formed on an inverted microscope (IX71; Olympus, Tokyo, Japan) by a 1,064-nm laser beam (ASF1JE01; Furukawa Electric, Tokyo, Japan) coming through an objective lens (CFI Apo TIRF 100× oil; Nikon, Tokyo, Japan). The cell, attached to a bead manipulated by the optical tweezers, was moved at a constant rate by actuators (SGSP-13ACTR-BO; Sigma Koki, Tokyo, Japan) attached to a microscope stage (Chukousya Seisakujo, Tokyo, Japan). Movies were recorded at 50 or 500 frames/s by a high-speed charge-coupled device (CCD) camera (LRH2500XE-1; DigiMo, Tokyo, Japan) and analyzed by using ImageJ 1.44p (http://rsb.info.nih.gov/ij/) and Igor Pro 5.05 (WaveMetrics, Portland, OR). To determine the relationship between the bead’s position relative to the trap center and the force exerted on the bead, various viscous forces were exerted on a polystyrene bead trapped with various laser powers by the optical tweezers by moving the stage at various speeds ranging from 0.012 to 2.0 mm s^−1^ (see Fig. S2 in the supplemental material) (51). The results did not depend on the vertical bead position, at least at 1.7 to 5.0 µm, suggesting that the shear stress need not be considered here. The trapping force, *F*, exerted on the bead was estimated from Stokes’ law, shown as *F* = 6πη*rv*, where η is the viscosity of the surrounding medium, *r* is the bead radius, and *v* is the speed of stage movement, and is plotted to the horizontal bead position (see Fig. S2B in the supplemental material). The trap’s stiffness as a function of distance between bead and trap center was proportional to the laser power used (see Fig. S2C).

The trapping force in the measurements was calculated with the standard curve at 104-mW laser power and the laser power used in the measurements. The trap’s stiffness at 200 nm from the trap center was calibrated as 0.40 pN nm^−1^ W^−1^ by measuring the position of the bead undergoing Brownian motion at a low laser power (52), in agreement with the above-described calibration based on Stokes’ law.

## SUPPLEMENTAL MATERIAL

Figure S1  Distributions of detachment forces under different pulling speeds. The averages indicated by filled triangles are integrated in Fig. 3C. Download Figure S1, PDF file, 0.1 MB

Figure S2  Calibration of trapping force. (A) Displacements of moving microscope stage and polystyrene bead trapped by optical tweezers. The stage was moved at 2.0 or 0.4 mm s^−1^, respectively, for the experiments whose results are shown in panels i and ii. (B) Trapping force as a function of the bead’s position relative to the trap center. The trapping force was estimated based on the measurements performed as represented in panel A, with the stage speeds ranging from 0.012 to 2.0 mm s^−1^. The shaded data points marked i and ii were derived from the corresponding measurements shown in panel A. (C) Trap stiffness divided by the laser power used as a function of the bead’s position relative to the trap center. The trap stiffness was calculated from the data in panel B. Download Figure S2, PDF file, 0.2 MB

Movie S1  Phase-contrast image of gliding cells of the wild-type strain. Download Movie S1, AVI file, 4.5 MB

Movie S2  Measurement of backward detachment. Real time. The bead, 1.5 µm in diameter, was manipulated by the optical tweezers and attached to the cell. The cell was detached from the glass surface at 00:27:00 by the displacement at 160 nm s^−1^. Download Movie S2, AVI file, 3.2 MB

Movie S3  Phase-contrast image of gliding cells of *gli521*(S859R) mutant. Download Movie S3, AVI file, 5 MB

Movie S4  Measurement of backward bond lifetime. The same measurement is shown in two different replay speeds, ×1 and ×1/10, as the first and the second scenes, respectively. The starved cell bound to the glass was displaced at 2.0 mm s^−1^ at 00:01:09. Download Movie S4, AVI file, 3 MB
