# Supplementary material for: Directed Binding of Gliding Bacterium, Mycoplasma mobile, Shown by Detachment Force and Bond Lifetime
Source: mBio. 2016 Jun 28;7(3):e00455-16. doi: 10.1128/mBio.00455-16 (PMC4937208; doi:10.1128/mBio.00455-16)
Supplement: Figure S1 — Distributions of detachment forces under different pulling speeds. The averages indicated by filled triangles are integrated in Fig. 3C. Download [file mbo003162865sf1.pdf]

FIG S1

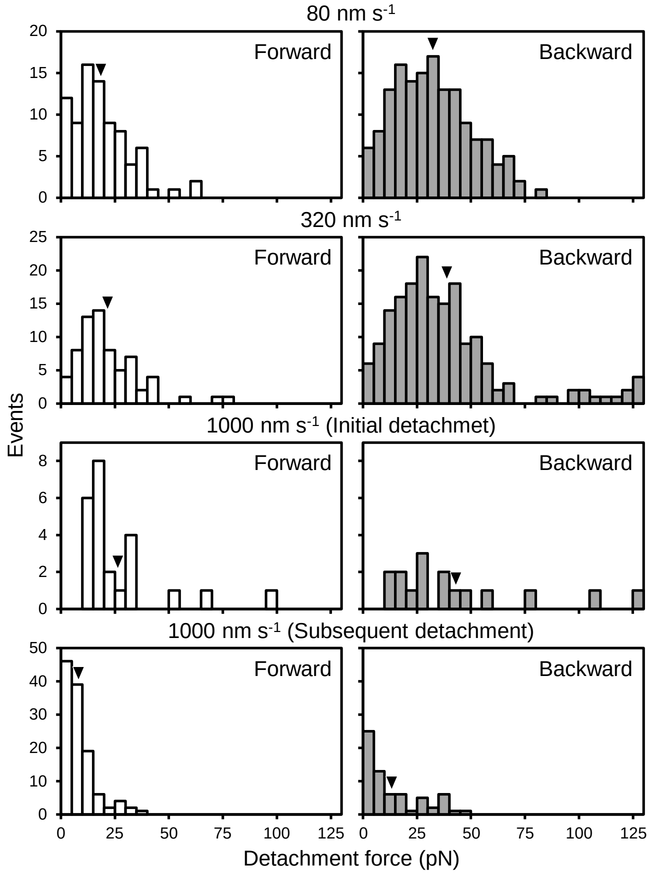

FIG. S1. Distributions of detachment forces under different pulling speeds. The averages indicated by filled triangles are integrated in Fig. 3C.
