# Supplementary material for: Directed Binding of Gliding Bacterium, Mycoplasma mobile, Shown by Detachment Force and Bond Lifetime
Source: mBio. 2016 Jun 28;7(3):e00455-16. doi: 10.1128/mBio.00455-16 (PMC4937208; doi:10.1128/mBio.00455-16)
Supplement: Figure S2 — Calibration of trapping force. (A) Displacements of moving microscope stage and polystyrene bead trapped by optical tweezers. The stage was moved at 2.0 or 0.4 mm s−1, respectively, for the experiments whose results are shown in panels i and ii. (B) Trapping force as a function of the bead’s position relative to the trap center. The trapping force was estimated based on the measurements performed as represented in panel A, with the stage speeds ranging from 0.012 to 2.0 mm s−1. The shaded data points marked i and ii were derived from the corresponding measurements shown in panel A. (C) Trap stiffness divided by the laser power used as a function of the bead’s position relative to the trap center. The trap stiffness was calculated from the data in panel B. Download [file mbo003162865sf2.pdf]

FIG S2

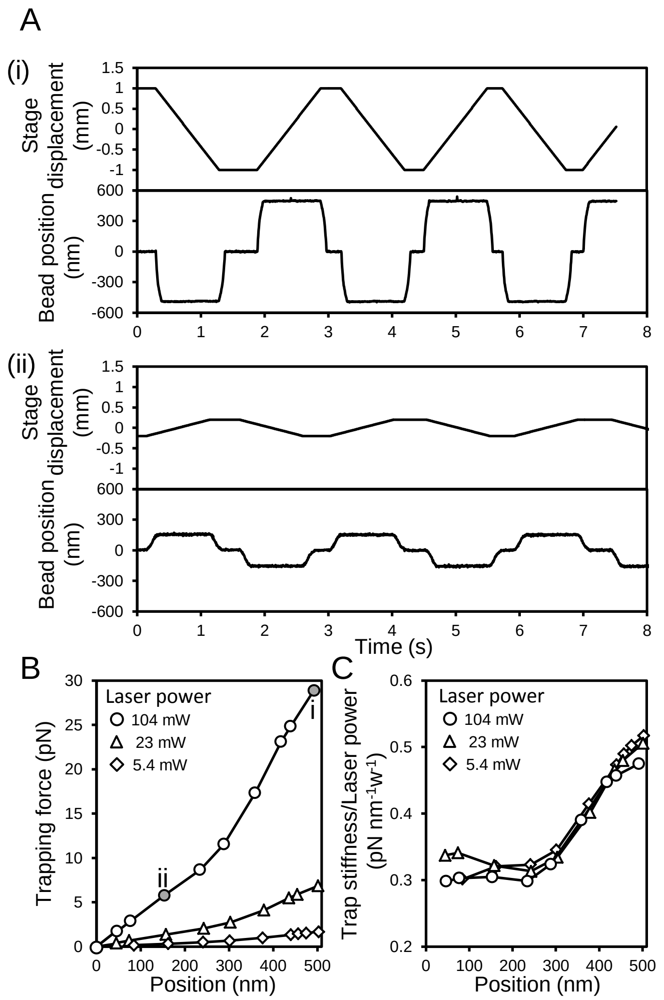

FIG S2. Calibration of trapping force. (A) Displacements of moving stage and polystyrene bead trapped by optical tweezers. The stage was moved at 2.0 and 0.4 mm s^{-1}, respectively, for (i) and (ii). (B) Trapping force as a function of the bead position from the trap center. The trapping force was estimated based on the measurements performed as represented in (A), with the stage speeds ranging from 0.012 to 2.0 mm s^{-1}. The toned data marked (i) and (ii) were derived from the corresponding measurements shown in (A). (C) Trap stiffness divided by the laser power used as a function of the bead position from the trap center. The trap stiffness was calculated from the data of (B).
